# Giant Ureteral Fibroepithelial Polyp Presenting As a Bladder Mass Resected Ureteroscopically: A Case Report

**DOI:** 10.5812/numonthly.4933

**Published:** 2012-12-15

**Authors:** Ali Momenzadeh, Farhad Sarrafzadeh, Mohammad-Hossein Nourbala, Amin Saburi, Zeinab Telkabadi

**Affiliations:** 1 Nephrology and Urology Research Center, Baqiyatallah University of Medical Sciences, Tehran, IR Iran; 2 Department of Internal Medicine, Faculty of Medicine, Kerman University of Medical Sciences, Kerman, IR Iran; 3 Chemical Injury Research Center, Baqiyatallah University of Medical Sciences, Tehran, IR Iran

**Keywords:** Neoplasms, Fibroepithelial, Ureter, Ureteroscopy, Urinary Bladder

## Abstract

Primary ureteral neoplasms are very rare and its prevalence is less than 1% of all genitourinary neoplasms. We report a symptomatic giant ureteral fibro epithelial polyp in adult women presenting as a bladder mass which was resected ureteroscopically and reported at the first time from Iran. Cystoscopy is growing use in the treatment of urinary tract lesions Cystoscopy can be used in large lesions in centers with experience rather than open surgery.

## 1. Introduction

Primary ureteral neoplasms are very rare in adults and are responsible for less than 1% of all genitourinary neoplasms (1). Only one-fifth of them are benign, and the mesodermal benign tumor such as Ureteral Fibroepithelial polyp (UFP) is more common (2). The UFP is usually located on the proximal portion of the ureter and is covered by a layer of normal urothelium (3, 4). generally speaking, it is not significantly symptomatic unless it makes obstruction (partial or complete) which characterized by obstructive or irritability symptoms such as supra-pubic or flank pain, hematuria (usually microscopic), frequency and dysuria (3). The releasing symptoms depend on polyp size and situation. Imaging diagnostic studies show ambiguous space occupying lesions commonly which was certainly diagnosed and treated by excisional biopsy (endoscopic or surgical) and histopathological report (5). We report a giant ureteral fibroepithelial polyp in adult patients representing a bladder mass which was resected ureteroscopically at the first time from Iran.

## 2. Case Presentation

**Figure 1:**
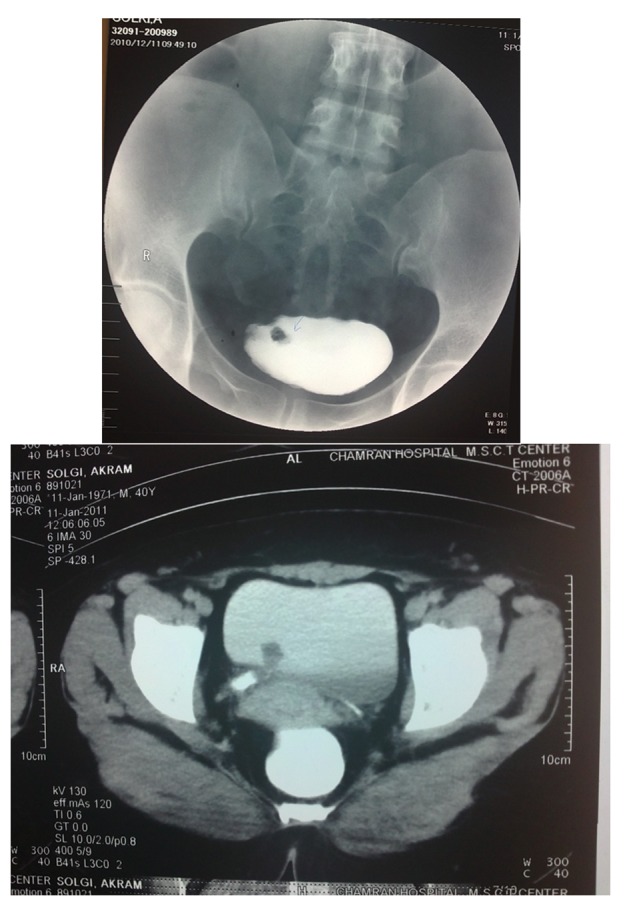
IVP Findings; A Small Round Shape Mass Near to Trigon Which Was Prolapsed Into Bladder

**Figure 2:**
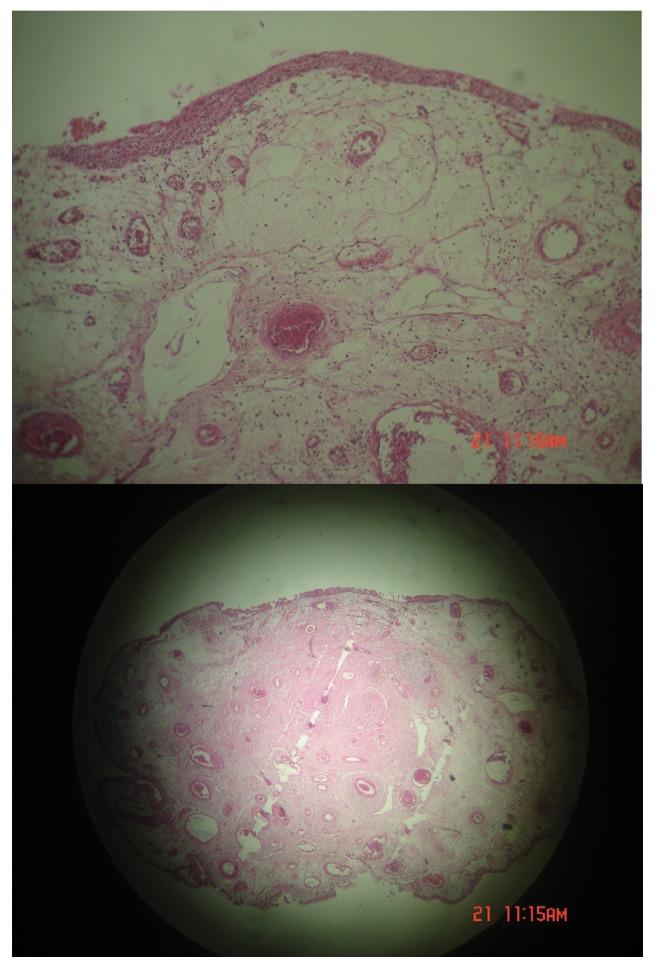
Pathologic Figures

A medical center diagnosed dysuria with obscure lower abdominal pain (during avoiding, specially) in a 44-year old female patient two weeks ago. Microscopic hematuria results from urine and blood sample laboratory analysis. She had no remarkable history of renal stone, urinary tract infections or any urinary stigmas. Physical examination has shown mild tenderness in right postvertebral angel without any other significant evidence. In hydronephrosis, a small effect has been diagnosed in the urinary bladder (US) which located on the right ureterovesical junction without any evidence of hydronephrosis. Computed tomography (CT) scan and IVP have revealed a small vegetative mass with 50×18 mm diameter situated near to trigon (Figure 1). Cystoscopy showed a pedunculated polyploidy tumor establishing on the right ureterovesical orifice and then the lesion was resected completely. Histopathology report was compatible with polyploidy cystitis without malignant components. Forty days later, the patient was admitted in this center due to dysuria and right flank pain. Except microscopic hematuria in urine cytology, another laboratory result consisting blood biochemical data were in normal ranges and urine cultures were negative. Renal US displayed moderate to severe hydronephrosis. She was undergone Re-Cystoscopy with diagnosis of urinary bladder mass. Cystoscopy showed a mass with a lobulated appearance sticking outside of the right vesicoureteral orifice. The fragmentally mass resection revealed the base of the tumor (90 mm in length and 10 mm in diameter) situated in the lower portion of ureter. The polyp stalk was coagulated and excised with claspers forceps and then the whole right ureteroscopy was assessed perfectly. Double-J stent was resided to prevent the ureteral stricture and periodically was reexamined by ultrasound or IVP until removal (3 weeks later). Histopathological analysis and retrospectively previous specimen re-assessment have confirmed a ureteral fibroepithelial polyp (Figure 2). There were no evidences of polyp recurrence or ureteral stenosis in 12 months after the double-J stent discontinuation and the patient was asymptomatic.

## 3. Discussion

Neoplasms which originated from ureter are rare and commonly malignant. However, UFPs are the most common non-malignant neoplasms; they are still rare clinically and primarily seen in males and on the left side (1). UFPs grossly seem as finger-like projection originating from the sub-mucosa of the ureter which attached to a single base (6). UFPs are composed of vascular and fibrous tissue covered with normal transitional epithelium (7). The clinical presentations are different depending on lesion location in ureter (1). UFP can be lengthened enough prolapsed into the bladder cavity and it can induce surgeons' aberrant (8). Management plan has different location and complication (5). Endoscopic plan of large UFPs is an acceptable treatment option with minimal adverse complications and durable treatment outcomes (5, 6, 9). Open resection approved a ureteral polyp originating from the lower ureter elongated into the bladder, which mimicked vesicle mass (7, 8).

We report a case of large UFP presenting a vegetative bladder mass excised ureteroscopically. Reviewing the English literature illustrated multiple reports of UFP, but ureteral fibroepithelial polyp elongated into the bladder cavity was rare which mimicked bladder mass resected ureteroscopically (3, 8-15). UFP was not reported from Iran and it was the first report. The authors recommend further assessment for any mass juxtaposed vesicoureteral junction and also suggest ureteroscopically approach to distal UFPs.
